# Piloting the role of a pharmacist in a community palliative care multidisciplinary team: an Australian experience

**DOI:** 10.1186/1472-684X-10-16

**Published:** 2011-10-31

**Authors:** Safeera Y Hussainy, Margaret Box, Sandy Scholes

**Affiliations:** 1 Centre for Medicine Use and Safety, Faculty of Pharmacy and Pharmaceutical Sciences, Monash University, 381 Royal Parade, Parkville 3052, Melbourne, Australia; 2 c/o Calvary Health Care Bethlehem, Kooyong Road, Caulfield South, Melbourne, Australia; 3 PO Box 205, Sandringham, Melbourne, Australia

## Abstract

**Background:**

While the home is the most common setting for the provision of palliative care in Australia, a common problem encountered here is the inability of patient/carers to manage medications, which can lead to misadventure and hospitalisation. This can be averted through detection and resolution of drug related problems (DRPs) by a pharmacist; however, they are rarely included as members of the palliative care team. The aim of this study was to pilot a model of care that supports the role of a pharmacist in a community palliative care team. A component of the study was to develop a cost-effective model for continuing the inclusion of a pharmacist within a community palliative care service.

**Methods:**

The study was undertaken (February March 2009-June 2010) in three phases. Development (Phase 1) involved a literature review; scoping the pharmacist's role; creating tools for recording DRPs and interventions, a communication and education strategy, a care pathway and evidence based patient information. These were then implemented in Phase 2. Evaluation (Phase 3) of the impact of the pharmacist's role from the perspectives of team members was undertaken using an online survey and focus group. Impact on clinical outcomes was determined by the number of patients screened to assess their risk of medication misadventure, as well as the number of medication reviews and interventions performed to resolve DRPs.

**Results:**

The pharmacist screened most patients (88.4%, 373/422) referred to the palliative care service to assess their risk of medication misadventure, and undertook 52 home visits. Medication reviews were commonly conducted at the majority of home visits (88%, 46/52), and a variety of DRPs (113) were detected at this point, the most common being "patient requests drug information" (25%, 28/113) and "condition not adequately treated" (22%, 25/113). The pharmacist made 120 recommendations in relation to her interventions.

Fifty percent of online survey respondents (10/20) had interacted 10 or more times with the pharmacist for advice. All felt that the pharmacist's role was helpful, improving their knowledge of the different medications used in palliative care. The six team members who participated in the focus group indicated that there were several benefits of the pharmacist's contributions towards medication screening and review.

**Conclusions:**

The inclusion of a pharmacist in a community palliative care team lead to an increase in the medication-related knowledge and skills of its members, improved patients' medication management, and minimised related errors. The model of care created can potentially be duplicated by other palliative care services, although its cost-effectiveness was unable to be accurately tested within the study.

## Background

Approximately 50 to 90% of cancer patients, and 9 to 16% of non-cancer patients, are referred into palliative care services per 100, 000 population per year in Australia [1]. Palliative care service provision occurs in three settings: in the community, in designated palliative care or hospice facilities, and within acute care hospitals. Community settings include the patient's home or a community living environment such as an aged or supported care facility [2]. Home is the most common setting, where approximately 70 to 80% of patients receive palliative care [3], with a significant proportion choosing to die at home [2].

A common problem encountered in the home is the inability to adequately manage medications, which is a significant issue given that they are used to treat symptoms experienced by the patient, such as pain. Inadequate medication management often stems from poor medication-related knowledge and understanding, in other words, health literacy [4,5], and can lead to non-concordance with medication regimens - medications not being taken, taken the wrong way or in the wrong dose, which can result in substantial treatment issues for the patient with impacts on their quality of life. The palliative care population has been identified as one of the groups who are at the highest risk of medication misadventure and adverse events, and consequently hospital admissions [6]. In particular, this is an unfavourable outcome when high risk medications such as opioids are involved, which are strong in potency, required to be taken chronically and have a high incidence of significant side effects such as constipation, drowsiness and nausea.

Further compounding this problem is the lack of consumer-oriented clinical information on medications. This is especially important as medications that patients needing palliative care take are for reasons and doses different to that described in information produced by pharmaceutical manufacturers, known as consumer medicine information (CMI) in Australia. This information is brand-specific and is based on the manufacturer's approved usage of the medication. However, as it is too complex for patients and carers to understand and does not cater to the educational needs of those from non-English speaking backgrounds [5,7], patients and carers are left with no choice but to search other primary and secondary sources of information such as the Internet [7], that are not pre-assessed to determine their level of evidence, and could result in patients opting for treatments that are not appropriate and potentially cause harmful outcomes.

Such problems also result in indirect expenditures, such as psychological, social and economic burdens on patients, carers and the healthcare system, which are generally much greater than the costs of pharmacological and non-pharmacological treatments [2,8]. The palliative care health team is in an ideal position to address and curb these issues in a timely manner, so that their effects are minimised. Quality of care is enhanced when patients are able to be treated by a team that comprises a variety of health professionals, each with their own set of knowledge, skills and experience. This includes, but is not limited to, specialised medical and nursing staff, and allied health professionals such as physiotherapists, music therapists, pastoral care workers, occupational therapists, pharmacists and counselling professionals [1].

Pharmacists, however, are not widely recognised as members of the palliative care health team [2,9,10]. The pharmacist is an integral team member whose contributions can potentially improve the patient's medication management and reduce their risk of non-concordance and hospital admissions [11]. Hussainy (2007) has indicated, however, that the pharmacist has other roles beyond medication management [8]. In a recent study by Wilson (2011), the majority of pharmacists' recommendations were accepted by physicians, and most patients achieved the desired clinical outcome - improvement or resolution of the presenting symptom. The pharmacist's recommendation, the recommending pharmacist, and closer proximity to death were found to be significant predictors of achieving the desired clinical outcome [12]. This highlights that acceptance and acknowledgement of the pharmacist's expertise in medication management is essential for supporting their role in a team.

In Australia, the role of a pharmacist in a community multidisciplinary palliative care team had not previously been piloted and a model of care to support and optimise this had not been developed and trialled. The overall aim of this study therefore was to develop a model of care that supports the role of a pharmacist as a member of a community palliative care multidisciplinary team (called "the team" hereon) and is potentially cost-effective, to improve outcomes for patients at home and their carers. Specifically, the pharmacist's role was to assist in improving knowledge of medications and their management for the health professionals within the team, and for patients and their carers. A major thrust was to minimise medication misadventure and, where possible, to reduce hospital admissions and lower costs to the service system.

The team operated within a service that is a member of the Southern Metropolitan Region Palliative Care Consortium (SMRPCC, http://www.smrpalliativecare-consortium.org.au) in Melbourne, Australia. The service provides specialist palliative care and neurological care in the community, covering a population base of over 500, 000. Patients may be referred by their carers, general practitioners (GPs), other health care professionals, external agencies, or by self-referral. The service admits over 500 new patients each year, with an average of 180 patients on the program at any one time. Patients' ages range from 1-90+ years (median age 65+), and approximately 85% have a malignant diagnosis and 15% non-malignant, including end stage Motor Neurone Disease. Prior to the integration of the pharmacist (known as the "Project Pharmacist" hereon) in to the team, it provided specialist medical, nursing, allied health, pastoral care, and bereavement services with the support of trained volunteers.

## Methods

The study was undertaken from February 2009 to June 2010 in three key phases described below. Approval by the ethics committee at the palliative care service was not required as the work undertaken in this study was deemed to be part of an ongoing quality improvement service to patients and their carers/families.

### Phase 1: Development

The objective of this phase was to develop various activities, namely pathways/strategies and tools, informed by information in the literature, which the Project Pharmacist (S.S) could use and follow when collecting data, collaborating with other members in the palliative care team, and interacting with patients and carers.

#### Literature review

A literature review was commenced in this phase and continued throughout the study, to provide a framework for the Project Pharmacist's role. Five areas were investigated, the results of which are reported elsewhere [5]: medication reviews; prescribing in palliative care; interventions; patient/consumer information; and patients at risk of non-concordance.

#### Project Pharmacist's role

Based on the findings of the literature review and with advice and support from the following people, the Project Pharmacist's role was developed:

• Project Team (which was separate to the team above and comprised an Allied Health Manager, Director of Clinical Services, Chief Pharmacist who provided inpatient services, Team Leaders and Project Manager - M.B)

• External Evaluator (S.H) who is a pharmacist with palliative care expertise

• Steering Committee that was established

• Key stakeholders from various organisations/networks

#### Tools

To assist the Project Pharmacist in undertaking their role, various tools were developed/adapted, based on the findings of the literature review.

The Medication review screening tool (MRST, see additional file 1) was developed to help determine if the patient is 'at risk' of medication misadventure and record other information such as diagnosis, allergies/adverse drug reactions, and renal and hepatic function. This tool was based on the "Home Medicines Review (HMR) Referral" [13], which the general practitioner (GP) uses to facilitate a HMR for a patient in their home, as well as other literature [14-16]. However, the MRST was developed to capture other risk factors than those in the HMR referral, as the majority of patients being palliated would be eligible for a HMR based on the criteria in the HMR referral.

The Intervention tool (see additional file 2) was adapted from "D.O.C.U.M.E.N.T (Drug selection, Over or underdose prescribed, Compliance, Untreated indications, Monitoring required, Education or Information, Non-clinical, and Toxicity or adverse reaction) for Medication Review" [17], to assist the Project Pharmacist in documenting drug related problems (DRPs) and recommendations made to resolve them (both descriptions and classifications of these).

#### Project Pharmacist's Activity Database

A database was developed so that the Project Pharmacist could log her activities and enter in data collected using the MRST and Intervention tool.

#### Care pathway

A care pathway was developed to assist members of the team in better managing patients.

*Where a patient was referred to the service*, this pathway involved a nursing admission assessment being undertaken first, prior to the patient's case being presented at the weekly team meeting. Based on knowledge gained at the team meeting, the patient's history and referral documentation, the Project Pharmacist then conducted an initial medication screening using the MRST. If the MRST found the patient to be 'at risk' of medication misadventure, the Project Pharmacist was available to conduct a medication review.

The Project Pharmacist was available to undertake the medication review on admission, when visiting the patient in their home with the Community Nurse at admission (at discretion of one of the Team Leaders), or later in the patient's home if deemed appropriate by a team member.

*Where a patient was discharged from the inpatient service (hospital) to home and referred to the Project Pharmacist*, she visited the patient prior to discharge and informed them that a home visit could be organised within 7-10 days after discharge. In this scenario, the Project Pharmacist performed an outreach medication review.

*Where a patient was discharged from another hospital to home and referred to the Project Pharmacist*, she contacted the patient or their carer to offer a home visit within the same time frame and conduct an outreach medication review.

All referrals were made using a standardised Referral Form that was developed.

Following medication review by the Project Pharmacist, the patient's treatment plan was updated and the Project Pharmacist provided feedback to the team, the patient's GP, and community pharmacy as necessary in the form of a Medication Review Report.

#### Evidence based patient information

Patient information leaflets (PILs) on the following nine medications used in palliative care in were developed: clonazepam; cyclizine; fentanyl; gabapentin; hydromorphone; metoclopramide; morphine; oxycodone; and pregabalin.

The morphine leaflet was piloted for face and content validity by a sample of patients and carers, doctors, the team and volunteers from the palliative care service (including an ex-pharmacist and people from a non-English speaking background). Based on the information, most patients/carers felt they fully/mostly understood how to take the medication and what to do if side effects occur. Some patients felt they only had a basic understanding, and were not confident, of what to do for side effect management. As these aspects could be explained to the patient/carer by the Project Pharmacist (e.g. during a home visit), the format and content was kept consistent for the remainder of the PILs.

Four of the PILs (morphine, fentanyl, hydromorphone and oxycodone) were translated in to seven languages (Arabic, Greek, Italian, Sinhalese, Traditional chinese, Vietnamese and Hindi) frequently encountered in the SMR Palliative Care Consortium. This was done by three translators (accredited by the National Accreditation Authority for Translators and Interpreters, NAATI) who are specialised and experienced linguists. The translated material was independently checked.

Audio (MP3) files of the morphine leaflet were also produced in English and in the seven other languages above.

The PILs and MP3 files were attached to websites (e.g. of the palliative care service, SMRPCC) for use by health professionals to assist patients and carers in understanding palliative care medications.

#### Communication strategy

A communication strategy for the Project Team, for within the palliative care service, and for key stakeholders, was developed. The Project Manager and Project Pharmacist met with key stakeholders (e.g. Pharmacy Guild of Australia, Pharmaceutical Society of Australia, General Practice Divisions Australia, Palliative Care Victoria) to discuss the project, how their organisation/network might be used to facilitate further communication via e-mail, newsletters and articles, and to provide, where relevant, education to their members at information sessions.

#### Education strategy

The Project Pharmacist developed and provided education to the palliative care service involved in the study, as well to as other palliative care services and health professionals (e.g. GPs) within the SMRPCC. Topics covered included neuropathic pain, enteral administration of medications and prescribing in the elderly and in renal and liver impairment.

Interprofessional education also occurred informally during daily interactions between the Project Pharmacist and other team members and formally at regular team meetings.

The Project Pharmacist also taught patients and carers to use a dose administration aid (DAA) for effective medication management.

### Phase 2: Implementation

The objective of this phase was to implement the activities discussed in Phase 1. Where necessary, adjustments were made to these based on feedback from the Project Team, External Evaluator, Steering Committee and key stakeholders. The Project Pharmacist was also clinically supervised, to a limited extent (because the Project Pharmacist had considerable experience and expertise in palliative care), by a Senior Medical Officer (doctor). Interaction between the Senior Medical Officer and Project Pharmacist involved, but was not limited to: joint community visits, particularly when complex issues were identified; follow up of patients'/carers' concordance to newly implemented medications/changes subsequent to this; education of patients/carers; advice regarding suitable drug formulations (e.g. for patients with swallowing difficulties); post-medication review discussions (when the Project Pharmacist had undertaken review separately); development of resource charts (e.g. benzodiazepine equivalence chart); auditing of patient's current medication regime (e.g. determining whether the medication list was recorded accurately/updated in the last month); and auditing of the process of requesting emergency medications from the GP (e.g. need for anticipatory prescribing to manage symptoms on a 24 hour "emergency" basis and need to have these medications available in the home).

### Phase 3: Evaluation

The objective of this phase was to evaluate the results of the activities implemented in Phase 2, in order to enable appropriate decision making and policy development by government and the palliative care service regarding the benefits of an ongoing role of a pharmacist in community palliative care services in to the future:

Evaluation of the study was grouped in to three categories: process; impact; and outcomes. This meant assessing the success of the process that underpinned each activity implemented in the study in Phase 2; the impact of each activity on the role of the Project Pharmacist in the palliative care team; and the clinical outcomes generated by each activity in terms of the palliative care team's knowledge and understanding of medications, the number of patients screened as well as medication reviews undertaken by the Project Pharmacist with a view to reducing DRPs, and the number of clinical interventions made.

In addition to using the three categories above, evaluation of the study was divided in to internal and external arms that are described below.

#### Internal evaluation

These three categories were studied not just during Phase 3, but also in earlier phases. During Phases 1 and 2 of the study, the Project Manager and Project Pharmacist regularly reviewed and revised its objectives and timeframes as necessary, in consultation with the Project Team and Steering Committee. The External Evaluator also reviewed all of the processes and documents discussed in Phase 1, including statistical data collation and analysis. As well, a 12-item survey was designed in Phase 1 and sent as an online link to other team members (n = 32) in Phase 2, to measure the number of times the Project Pharmacist had been contacted, the type of interactions and the considered value of the role within the team. Open-ended comments could also be made.

#### External evaluation

In Phase 3, the External Evaluator conducted a focus group with the team (not including the Project Pharmacist or Project Manager) to further determine the effectiveness of the study in terms of supporting the role of a pharmacist in a community multidisciplinary palliative care team. The focus group was conducted at the palliative care service, using a semi-structured guide, and was audio recorded.

The results of the internal and external evaluations were then combined and are shown in Table 1, according to the program levels and indicators (in descending order of level of evidence) adapted from Suvedi and Morford (2003) [18] and according to each Phase of the study in which they occurred.

**Table 1:** Combined results of internal and external evaluations of the study

Program Levels	Indicators	Results, including phase of the study and evaluation strategy in which this was demonstrated
End results	What long term changes occurred as a result of the project?	• Improved knowledge of how/where to access information/medications/management by the team (Phases 1 and 2, online survey; Phase 3, focus groups)
		• Increased knowledge of palliative care medications and their use (Phases 1 and 2, online survey; Phase 3, focus groups)
		• Development of PILs - available on website of palliative care service (includes translations and MP3 files) (Phase 3)
		• Care pathway developed (Phase 1)
		• MRST developed (Phase 1)
		• Toolkit for use by other palliative care services (Phase 3)

Changes in practice and behaviour	How did practice change as a result of project participation?	• Acceptance of the role of a pharmacist within a team (Phases 1 and 2, online survey; Phase 3, focus groups)
		• Use of medication sheets in nursing folder for chemotherapy and other medications (Phases 2 and 3)
		• Documentation and process of obtaining emergency medications - reviewed as part of audit (Phases 2 and 3)

Changes in knowledge, attitudes, skills and aspirations (KASA)	How did participants' knowledge, attitudes, skills and aspirations change as a result of project participation?	• Increase in knowledge and skills of team with respect to medications and their management, and on complexity of medication regimens (Phases 1 and 2, online survey; Phase 3, focus groups)

Reactions	How did participants and clients react to the project activities?	• Project Pharmacist accepted as an allied health professional within the team (Phases 1 and 2, online survey; Phase 3, focus groups)
		• Team very positive (Phases 1 and 2, online survey; Phase 3, focus groups)
		• Patients/carers accepting of the Project Pharmacist as part of the team (Phase 2)

Participants	Who participated and how many?	• Project Team, Steering Committee, External Evaluator, Team members, staff working at the palliative care service (all involved in consultation and communication strategies)
		• 380 patients were screened using the MRST (Phases 2 and 3)
		• 52 home visits to patients for medication review (Phases 2 and 3)

Activities	In what activities did the participants engage through the project?	• In-services to team (Phase 2)
		• Education and information sessions for SMRPCC members and health professionals working within this consortium and at another consortium (Phase 2)
		• Medication screenings using the MRST and medication reviews in patients' homes (Phase 2)
		• Conferences and seminars (Phases 2 and 3)
		• Consultation with key stakeholders (all Phases)
		• Newsletter articles (all Phases)

Inputs	Which personnel and other resources were used during the project?	• Project Team/External Evaluator/Steering Committee (all Phases)
		• Members of the team (all Phases)
		• Staff working at the palliative care service (all Phases)
		• Clinical supervision and support (Phase 2)
		• Volunteers - assistance with reviewing and critique of PILs (morphine) (Phase 2)
		• Liaison with outreach pharmacists, clinical trial pharmacists, key stakeholders (all Phases).

## Results

### Project Pharmacist's role

On review of the Project Pharmacist's Activity Database, it was demonstrated that the Project Pharmacist undertook the following seven major roles [8]:

1. Medication review

2. Education for patients/carers e.g. carer training on how to use DAAs and other delivery devices, such as nebulisers and oxygen concentrators.

3. Ensuring ongoing access to medications e.g. home delivery of medications in palliative care emergencies

4. Information provision/education for team members e.g. on off-label medications, oral chemotherapy or trial medications used by patients (this information was inserted into the patient's medical history and "working file" for all team members to access)

5. Consultation and collaboration with team members e.g. updating medication chart

6. Liaison with other health professionals (e.g. hospital, community and outreach pharmacists, GPs, palliative care nurses) to ensure continuity of patient care.

7. Symptom management protocol (e.g. on nausea and vomiting, constipation) implementation through in-services education.

In addition, the Project Pharmacist conducted a combined bereavement visit (with a social worker) for a patient's carer. The Project Pharmacist found the experience to be valuable as it provided the opportunity for interprofessional learning and development of this counselling skill.

### Impact of Project Pharmacist's role in the team

#### Online survey

A 63% response rate was achieved (20/32 team members). 50% of respondents (10/20) had interacted 10 or more times with the Project Pharmacist, during the past three months, for advice/assistance. Only 5% (1/20) had never interacted with the Project Pharmacist; this respondent was a non-clinical staff member who would have been unlikely to have needed this type of contact.

The majority of interactions were regarding information/clarification about medications (94.7%, 18/19), advice on what medications could be used (73.7%, 14/19) or advice on alternative medications (63.2%, 12/20). Assistance in decision-making when changing a medication was less commonly sought (47.4%, 9/19). Comments made by respondents also indicated that they accessed the Project Pharmacist for other aspects of medication management e.g. impact of medications on functional status, minimising medications.

With regards to whether respondents found the Project Pharmacist's role helpful, all (20/20) felt that it had improved their knowledge of the different medications used in palliative care, 90% (18/20) indicated that it had improved their knowledge of medication issues for palliative care patients, and 60% (12/20) thought it had changed their practice with sourcing and providing information to patients/families. One respondent commented: *"I have been able to provide better, needs-based care"*.

The majority of respondents (95%) said they would be more likely to discuss patients who had potential medication issues with the Project Pharmacist. When asked about how they found out information prior to the role of the Project Pharmacist within the team, the majority *"found it themselves and/or approached the Chief Pharmacist"*. The Chief Pharmacist indicated that the Hospital Pharmacy rarely received requests from the team for information on medications or medication management since the role of the Project Pharmacist was introduced, and as a result, had *"lessened the anxiety" *they had felt when providing advice for patients who were deemed to be part of an 'invisible ward' i.e. patients who they had not seen but it was expected that they were to provide information about their medications/treatments.

#### Focus group

Six team members participated in the focus group (1 Registrar [P1], 1 Occupational Therapist [P2], 1 Social Worker [P3], 3 Nurses [P4-6]). When asked what they perceived were the benefits of the Project Pharmacist's contributions towards medication screening and review, participants felt that she was readily accessible, approachable and knowledgeable, especially when called on for information during home visits: *"Great resource and help especially when there's a problem in the home, can pick up the phone and ask [her] e.g. unfamiliar drug, doses, calculations" *(P4).

One participant (P5) stated that the Project Pharmacist was *"confidence-building...helping give better service and better outcomes for patient[s] especially because [they] know [the] patient's background"*. Another (P3) commented that the Project Pharmacist: *"picked up on non-adherence and impact on behaviours"*, and *"made [me] question about why it's not always necessary to prescribe emergency meds, need to be more client-centred, emergency meds cost money. GPs aren't always receptive about emergency meds, and saying you've chatted to the pharmacist helps with adding credibility/authenticity"*.

Participants perceived that the Project Pharmacist's role had few limitations, mainly being that that it made them ignore their own learning as they tended to rely on her. When asked what areas required improvement, participants believed that she should have been given more opportunities to attend home visits and increased time to dedicate to the role. Participants also felt it was important that the Project Pharmacist *"was participating in team meetings in order to pick up on things and follow up - this is a key component and would need to be maintained in to future services"*.

Aside from attending team meetings, participants thought that to sustain this role in a community multidisciplinary palliative care team, a future pharmacist needs to be *"experienced and [have] a palliative care approach to the way they think and interact with families"*. Participants believed that the model of care piloted in the study was sustainable (and needed) as it eliminated a gap in care that existed, was an *"essential component of interdisciplinary management for patients being palliated at home"*, where each component was recognised as being important, and *"provided a quality service for patients and families" *that involved reducing the risk of medication misadventure and its potential consequences:

*"[It's] about providing a safe service and acting quickly e.g. on what GPs are prescribing, not necessarily what's wrong with our service"*.

*"Every community service should have a pharmacist"*.

*"Can expand positives to other teams, huge loss not to have a pharmacist"*.

Finally, to further ensure sustainability, participants indicated that the gap between hospital to community care needs to be bridged but were unsure about how this could be done considering that the Project Pharmacist had been *"proactive and education-focussed"*.

### Impact of Project Pharmacist's role on clinical outcomes

#### Medication reviews

The Project Pharmacist Activity Database showed that screening for patients at risk of medication misadventure was conducted for the majority of patients (88.4%, 373/422) referred to the palliative care service, using the MRST. 380 MRSTs were conducted from April 2009-March 2010, during which the following problems were frequently detected by the Project Pharmacist:

• 83% (316) took 5 or more medications, or more than 12 doses of medications per day.

• 77% (294) took medication requiring monitoring, had a narrow therapeutic index or are high risk.

• 62% (238) had other co-morbidities

• 55% (208) were recently discharged from hospital

• 59% (224) were attending different healthcare providers

Allergies/adverse drug reactions were recorded routinely since October 2009 in 185 MRSTs; 24% (44) reported no known allergies/adverse drug reactions; 31% (58) had none recorded; and 45% (83) had data recorded. Similarly, renal function was routinely recorded since December 2009 in 116 MRSTs. There was data available for 49% (57) of patients.

Fifty two home visits were undertaken by the Project Pharmacist from June 2009-March 2010, and these were done on a "needs basis" after consultation with other team members. Approximately 6 home visits per month were undertaken by the Project Pharmacist, and on average, a home visit took 54.5 minutes. Where there was no need for a home visit, sometimes care was coordinated by the Project Pharmacist over the telephone, or by other means, which took substantial time; this was not counted as a home visit.

Medication reviews were conducted at the majority of home visits (88%, 46/52), and a report of the findings was generated for all patients seen by the Project Pharmacist, a copy of which was compiled into the patient's medical history and sent to their GP, community pharmacist and other health professionals involved in the patient's care if appropriate.

The Project Pharmacist perceived the medication reviews undertaken at home visits to have had a significant impact on patients' care, through reassurance and education around medication management for patients/carers. She felt that the impact of medication reviews on team members was that they were provided with an updated medication list for all patients, and therefore knew exactly what medications the patient was currently taking.

#### Interventions

The Project Pharmacist made 113 interventions based on the same number of DRPs that were detected, and the most common DRPs were "patient requests drug information" (25%, 28/113) and "condition not adequately treated" (22%, 25/113). The Project Pharmacist made 120 recommendations in relation to her interventions, and the most common types were "referral to the prescriber" (35%, 42/120) and "education/counselling session" (30%, 36/120).

It could not be estimated, however, as to how many recommendations arising from medication reviews were accepted by prescribers, as the Intervention tool was used infrequently, and there was little correspondence from GPs in response to the Medication Review Reports generated by the Project Pharmacist. A lot of the time though symptom management issues were managed by doctors at the palliative care service and scope for GP involvement was small; the purpose of the Medication Review Report was mainly to keep the GP up-to-date with the patient's progress. Also, in many cases the Medication Review report only recorded the patient education activities undertaken by the Project Pharmacist, where no GP intervention was required.

#### Toolkit

A toolkit was developed to assist other palliative care services in developing their own model of care, incorporating the role of a specialist pharmacist in to the team. Four options for sustainability were provided, as well as some "rough" costings for this role in to the future. Pathways/strategies and tools that were developed in this study, such as the care pathway, MRST, Intervention tool and PILs, were also included in the toolkit.

## Discussion

This study is the first in Australia to demonstrate that the inclusion of a pharmacist in a community palliative care multidisciplinary team: assists in increasing the knowledge of team members, with respect to medications used in palliative care and their management; leads to improved knowledge of potential problems with medications and how to manage them and to a change in practice for the benefit of patients; enables ongoing education and support from the pharmacist to the team members; allows for in-service education to be provided, as and when required; assists in improving contacts with the GPs and palliative care service for the benefit of the patient and their carer; and assists the patient and carer to better understand the medications prescribed.

In particular, the study led to a significant increase and improvement in knowledge of medications and their use by nursing staff. However, this result may in part be influenced by the role of the Senior Medical Officer in the team and their contribution to patients' medication management in concert with that of the Project Pharmacist's. Certainly, improving health professionals' knowledge of, and skills and confidence in medication utilisation has been recognised as key strategy in preventing medication misadventure and improving patient safety [19,20], especially in older and non-English speaking persons [21]. In line with recommendations made by Westberg and Sorensen [21], the Project Pharmacist considered the impact of language barriers when working with patients and carers to optimise their medication management. PILs for four medications in seven languages other than English were also designed for this purpose; however, were only developed in the final phase of the study and thus were not utilised by the Project Pharmacist. The PILs can potentially be used to assist medication management in community palliative care settings and further research is required to determine their face and content validity from the consumer perspective and whether they meet their educational, language and visual needs [22,23].

The study findings in terms of number of DRPs detected (113) by the Project Pharmacist are also comparable to those reported by Westberg and Sorensen [21]. In their study, the pharmacist detected 186 DRPs; these were greater among non-English speaking patients (31%) than English speaking patients (12%), and drug therapy outcomes improved by 24% once the pharmacist joined the team of clinic providers [21]. In another study by Needham, Wong and Campion, community pharmacists' interventions' in palliative care were assessed for significance [24]; in contrast, the study reported here did not assess the significance of the Project Pharmacist's interventions but rather, looked at the number of recommendations made (120) in relation to her interventions (113), which were readily accepted by doctors and other health professionals in the palliative care team. These results confer with those reported by Wilson where the majority of the palliative care pharmacist's interventions were accepted by physicians [12].

The study presented here is also the first to have reported the utility of a screening tool (MRST) in the palliative care population and therefore the first to present data on the number of patients screened (373/422) for being 'at risk' of medication misadventure. The number of home visits made by a pharmacist to undertake medication reviews for patients requiring palliative care (46/52) is also new data that is currently not available in the literature. This tool and measurement outcome, respectively, can reasonably be used in future studies where a pharmacist is involved, in addition to assessing the achievement of clinical outcomes such as symptom control [12,25] and an improvement in patients' and carers' medication related knowledge and understanding [26].

Although the timeframe of the study and the capacity to measure benefits was limited, it was recognised that the inclusion of a pharmacist could also lead to: an increase in confidence of patients/carers in the use of medications; an increase in medication concordance; a reduction in medication errors made by both health professionals and patients/carers; and reduced hospital admissions due to medication errors being averted by a comprehensive patient screening and medication review process, recognising that hospital admissions are sometimes required to improve medication and/or symptom management and can, ultimately, prevent future admissions as the patient's condition deteriorates. This is in line with the findings from a study by Ajdukovic et al, who found that pharmacist elicited medication histories for 'at risk' groups in the Emergency department can reduce medication misadventure and errors [27].

Specifically, this role in the present study alleviated the demand on the hospital pharmacists by having many of the team's queries regarding medications or medication management issues answered by the Project Pharmacist. This was a saving in time and money to this area of hospital operations. However, this also could not be quantified as previous requests for assistance varied in number and complexity. 'Interruptions' to the usual role of the hospital pharmacists also varied depending on whether they were in the pharmacy at the time of the request and so information was on hand, or whether they were on a ward and had to leave the task they were undertaking and return to the pharmacy to seek information to assist.

Importantly, this study attempted the development of a potentially cost-effective model of care (incorporating various pathways/strategies and tools), which has not been previously done locally or internationally. The care pathway provided a focus and framework for the Project Pharmacist, the team and the palliative care service, and is freely available to other palliative care services across Melbourne, Victoria for duplication via the toolkit that was developed. This toolkit should be added to the Australian Government Department of Health website for use by community palliative care services across Australia, and if appropriate, to international government websites. Indeed, its encompassing tools became familiar within the study community palliative care service and referrals increased. All patients were screened by the Project Pharmacist on admission to the team, to evaluate triggers for home visits and a more extensive medication review.

Although the development of a cost-effective model of service delivery was a component, this premise was unable to be achieved within the scope of the study because the structure of each palliative care service is so different. The community palliative care service was aided by the existence of a hospital pharmacy department that had the capacity to provide back-up to the Project Pharmacist. The pharmacy department and Project Pharmacist complemented each other. This additional support is not available in most community palliative care services. Further, a lack of pharmacists with the high level of knowledge and skills relevant to palliative care of the Project Pharmacist engaged for this study are unlikely to be able to be duplicated, at this point in time, in other community palliative care services.

There is the potential, however, to duplicate the model and make a saving on the employment costs by sharing a community pharmacist among a group of palliative care services. This would aid the potential for a cost-effective model because of the reduction in time spent with families once the pharmacist was involved; a lessening in the prescribing of unnecessary and/or ineffective medication; and an improvement in the overall knowledge of the other health practitioners and patients and families in recognising adverse effects of medicines and thus anticipating medication and treatment needs, with a subsequent improvement in the care and support of patients and their families.

There would need to be the engagement of more than one pharmacist in one service to more accurately predict a cost-effective model. This study could therefore, more realistically, be viewed as a scoping or feasibility study.

Further to the above, the model of care is not a 'one size fits all' paradigm and should be clearly seen in the context of location and requirements. A pharmacist employed in a single palliative care service, for example, may have a different role and structure from other employment models e.g. compared with employment by a palliative care consortium or within a division/network of GPs. Nevertheless, below are examples of recommendations that have been made to sustain the model of care; some or all of these could be considered by policy makers, palliative care services, divisions/networks of GPs, community/specialist pharmacists and pharmacy peak bodies in other states of Australia and overseas.

### Potential future of the role of pharmacists within palliative care services

As a result of this study, a number of recommendations and suggestions have been made that would assist decision-making on the efficacy of including a pharmacist within community palliative care services. These include recommendations regarding funding, region-wide or consortia collaborative arrangements and inclusion of a pharmacist within divisions/networks of GPs.

It was considered that funding should be made available for a specialist pharmacist to be placed in community palliative care services within Melbourne, Victoria. This would assist continuation of the 'value-add' and lessons learned from this feasibility study across the sector. Inclusion in the palliative care service would provide timely access to expertise, education and advice as well as improve patient medication management and symptom control.

Both government and palliative care services themselves should consider how this might be achieved. An agreement between palliative care services could be negotiated within the palliative care consortia or regions to fund a pharmacist position to support the services to improve medication management for their patient group and also knowledge of palliative care medications and their management within their community palliative care teams. A pharmacist placed within a consortium would need to incorporate all the skills required within a single service, but be broadened out in terms of actual tasks undertaken and service operation. This would require the capacity to manage the varying demands of the service within a region, and the compilation of an education information portfolio, including online access to information and support, to assist the various demands upon the time and knowledge of the pharmacist.

Alongside this, the pharmacist would need to remain up-to-date with changing referral processes and their relevance to palliative care patients. An essential component of such an arrangement would have to be a willingness by both the services and the pharmacist to be mobile. By its nature, the role of a pharmacist across a consortium or region would be less hands-on than one placed within a single service.

To aid improvement in symptom management and control by community palliative care services, there should be an extension of, the current limited funding of Outreach Pharmacy Services within public hospitals and it should ensure that palliative care patients are included within their scope of operation. Outreach Medication Reviews are available at major metropolitan hospitals in Victoria for patients at risk of medication misadventure after discharge from hospital. The Outreach Pharmacist provides counselling on medication and disease state management in the patient's home or specialist outpatient clinic. After a home visit, a report is sent to the patient's GP and community pharmacist. The extension of the capacity of the Outreach Pharmacy Services could provide an avenue for palliative care patients to obtain a medication review in a timely manner after discharge from hospital.

Divisions/networks of general practice should also consider incorporation of a specialist palliative care pharmacist within their division/network. The pharmacist's role would be different if located within a division/network of GPs, although much of the above would be required, and it could be assumed that the tasks would be broader than providing palliative care. Inclusion in this forum would allow for improved collaboration between hospitals and community palliative care services within these network areas.

At the same time, better links between peak pharmacy and palliative care bodies, with an accompanying improvement in communication between community pharmacists and palliative care services, would greatly assist in the support of patients and their families, especially in accessing, using and managing emergency just-in-case medications. This was recognised as an issue in this study and further research in to prescribing and dispensing of emergency medications is required to prevent duplication and wastage; specifically, to determine whether this results in an accompanying decrease in hospital admissions and what cost savings could be achieved by interventions resulting from HMRs for palliative care patients.

The development of a PIL for patients and carers on the use of emergency medications could also assist in alleviating concerns by patients and their families on how and when to take them. A generic document for use by palliative care services could be developed (with relevant local service adaptations incorporated as required) that can be sent to GPs, which includes all the relevant details required by the GP, including the form, strength, dose and cautions associated with emergency medications and who to contact if patient/family are unsure of how and what to do.

The development of a check list for nursing staff on the process of ordering emergency as well as regular medications, including follow-up processes and, an improvement in communication from palliative care services to GPs and patients regarding their use, would all aid better management of symptoms, and could lead to a reduction in hospital admissions and the capacity for patients to die at home if they desire. Anecdotally, however, it is reported that no matter how much planning and information is provided, some patients, carers and families can, understandably, panic and require outside assistance when faced with an emergency medication related problem.

### Limitations

Patients or carers were not surveyed with respect to their knowledge (to determine the impact of the Project Pharmacist on this), because often by the time the Project Pharmacist had screened medications and/or visited the patient, their condition had deteriorated to the extent that surveying would have been an imposition. Occasionally, patients had died before the Project Pharmacist had a chance to undertake a home visit.

## Conclusions

This study showed that the inclusion of a pharmacist in a community multidisciplinary palliative care team has great benefits for the team, patients and carers. There are seven major roles that a pharmacist can undertake, which can lead to an increase in the medication-related knowledge and skills of team members and thereby improve the medication and symptom management of patients and minimise medication errors. To support these roles a model of care is required and was created, incorporating various pathways/strategies and tools that can potentially be duplicated by other palliative care services. A larger, longitudinal study is required to accurately test whether this is a cost-effective model, incorporating a number of different *modus operandi*, for example, a pharmacist within a single service versus a pharmacist across consortia or one placed within a network/division of GPs.

From this feasibility study, it could be extrapolated that the cost-effectiveness of such a model would be demonstrated by the improved knowledge of medications and their use by both professionals and patients/families, and thereby better symptom identification and management, along with improved patient medication information and better communication between palliative care services, GPs, specialists and pharmacists. A foreseeable outcome would be fewer hospital admissions for the palliative care patient and a reduction in prescribing of unnecessary medications.

## List of abbreviations used

CMI: Consumer medicine information; DAAs: Dose administration aids; DRP: Drug related problem; D.O.C.U.M.E.N.T: Drug selection, Over or underdose; rescribed, Compliance, Untreated indications, Monitoring required, Education or Information, Non-clinical, and Toxicity or adverse reaction; GPs: General Practitioners; MRST: Medication review screening tool; NAATI: National Accreditation Authority for Translators and Interpreters; PIL: Patient information leaflet; SMRPCC: Southern Metropolitan Region Palliative Care Consortium.

## Competing interests

The authors declare that they have no competing interests.

## Authors' contributions

SH drafted the manuscript and made contributions to the conception and design of the study, as well as the collection (specifically for the focus group), analysis and interpretation of data. MB contributed to the design and coordination of the study; the acquisition (specifically for the online survey), analysis and interpretation of data; and revised the manuscript. SS also collected (specifically for the medication reviews and interventions), analysed and interpreted the data, and revised the manuscript. All authors have read and approved the final manuscript.

## Authors' information

SH graduated from Monash University (BPharm(Hons) in 2001 and PhD in 2007) and was appointed to the position of Lecturer in 2009 at the Faculty of Pharmacy and Pharmaceutical Sciences. She is an Early Career Researcher in Pharmacy Practice with research experience and expertise in professional practice, education and medication safety and use, particularly in the area of palliative care service delivery in the primary care setting. Her PhD research involved the identification of Australian community pharmacists' educational needs in palliative cancer care, and the subsequent development, delivery and evaluation of an educational program to meet these needs. This program will soon be adapted to suit a Masters in Pharmacy Practice program at Monash University for postgraduate students. SH also teaches palliative care to undergraduate students at Monash University along with other topics, and has a strong research interest in women's health.

MB was Executive Director of Palliative Care Victoria and on the Palliative Care Australia Council for several years. Her involvement with the palliative care sector has encompassed many roles. Since living in Thailand for some years, Margaret returned to Australia and managed a number of projects, including Development of a Model of Care for People Living with Motor Neurone Disease. She has recently left Australia to live overseas again.

SS has practical experience in palliative care in hospital and community settings, and has undertaken postgraduate studies in palliative care. She was a member of the expert group who updated the Therapeutic Guidelines: Palliative Care version 3, 2010. SS has had input in to two projects funded by the Australian Government Department of Health and Ageing, which were aimed at improving the medication management of palliative care patients by educating community pharmacists. She is currently assisting development of the palliative care unit for postgraduate study at Monash University.

## Pre-publication history

The pre-publication history for this paper can be accessed here:

http://www.biomedcentral.com/1472-684X/10/16/prepub

## Supplementary Material

Additional file 1 **Medication Review Screening Tool (MRST)**. This is the tool that was developed in the study for use by the pharmacist to assist them with screening patients who were admitted to the palliative care service to determine their risk of medication misadventure. Click here for file

Additional file 2 **Intervention form**. This is the tool that was developed in the study for use by the pharmacist to assist them with recording drug-therapy problems they detected in patients and recommendations they made to resolve those problems. Click here for file
