# Supplementary material for: Piloting the role of a pharmacist in a community palliative care multidisciplinary team: an Australian experience
Source: BMC Palliat Care. 2011 Oct 31;10:16. doi: 10.1186/1472-684X-10-16 (PMC3215169; doi:10.1186/1472-684X-10-16)
Supplement: Additional file 1 — Medication Review Screening Tool (MRST). This is the tool that was developed in the study for use by the pharmacist to assist them with screening patients who were admitted to the palliative care service to determine their risk of medication misadventure. [file 1472-684X-10-16-S1.PDF]

Additional file 1 – Medication Review Screening Tool (MRST)

Pharmacist in Community Palliative<br><br>Care Multidisciplinary Teams Project<br><br>**Medication Review Screening Tool**	Patient Details:<br><br>Name:<br><br>Address:<br><br>UR Number

Medication use

- □ Taking 5 or more medications, or more than 12 doses of medication per day
- □ Significant changes to medication treatment regimen in the last 3 months
- □ Started new medication in the last 4 weeks
- □ Taking medication not commonly used in primary care………………….
- □ High alert medication …………………………………………………….
- □ Use of alternative health care products
- □ Enteral feeding tube in-situ
- □ Symptoms suggestive of an adverse drug reaction
- □ Medication plan is not current
- □ Suspected non-adherence or inability to manage medication

Other

- □ Literacy or language difficulties, confusion/dementia or other cognitive difficulties
- □ Other co-morbidities or lifestyle practices [eg alcohol, tobacco, illicit drugs] which affect pharmacodynamics and pharmacokinetics……………………………………………………………
- □ Living alone or in Supported Residential Services, poor carer support or carer concerns

- ☐ Recent discharge from a hospital (in the last 4 weeks)
- ☐ Attending different healthcare providers eg, general practitioner, specialist

Diagnosis: ……………………………………………………………………………

Allergy/adverse drug reactions: ………………………………………………………

Renal function: ………………………………………………………………………

Hepatic function: ……………………………………………………………………

Comments:

……………………………………………………………………………..………………

………………………………………………………………………………………………

…………………………………………………………….................................

Signature: ……………………………………..Date: ………………………
