# Supplementary material for: Piloting the role of a pharmacist in a community palliative care multidisciplinary team: an Australian experience
Source: BMC Palliat Care. 2011 Oct 31;10:16. doi: 10.1186/1472-684X-10-16 (PMC3215169; doi:10.1186/1472-684X-10-16)
Supplement: Additional file 2 — Intervention form. This is the tool that was developed in the study for use by the pharmacist to assist them with recording drug-therapy problems they detected in patients and recommendations they made to resolve those problems. [file 1472-684X-10-16-S2.PDF]

Additional file 2 – Intervention form

The pharmacist would insert a free text description of the drug related problem (DRP) and their recommendation to resolve it, and use the categories below to classify the DRP and recommendation.

Pharmacist in Community Palliative Care Multidisciplinary Teams Project

Intervention tool

Description of Drug Related Problem	Classification of Drug Related Problem	Description of Recommendation	Classification of Recommendation

Classification of Drug Related Problems

D Drug selection	D1 Duplication<br>D2 Drug interaction<br>D3 Wrong drug<br>D4 Incorrect strength<br>D5 Inappropriate dosage form<br>D6 Contraindications apparent<br>D7 No indication apparent<br>D8 Other drug selection problem
O Over or under-dose	O1 Prescribed dose too high<br>O2 Prescribed dose too low<br>O3 Incorrect or unclear dosing instructions<br>O0 Other dose problem
C Compliance	C1 Taking too little<br>C2 Taking too much<br>C3 Erratic use of medication<br>C4 Intentional drug misuse (Including OTCs)<br>C5 Difficulty with dosage form<br>C0 Other compliance problem
U Under-treated or Untreated indication	U1 Condition not adequately treated<br>U2 Condition untreated<br>U3 Preventive therapy required<br>U0 Other untreated indication problem
M Monitoring required	M1 Laboratory monitoring<br>M2 Non-laboratory monitoring<br>M0 Other monitoring problem
E Education or Information	E1 Patient requests drug information<br>E2 Patient requests disease management advice<br>E0 Other education or information problem
N Not classifiable	N0 Clinical interventions that cannot be classified under another category
T Toxicity or adverse reaction	T1 Toxicity, allergic reaction or adverse effect present

Classification of Recommendations

A change in therapy	R1<br>R2<br>R3<br>R4<br>R5<br>R6<br>R7	Dose change<br>Drug change<br>Drug formulation change<br>Drug brand change<br>Dose frequency/schedule change<br>Prescription not dispensed<br>Other changes to therapy
A referral required	R8<br>R9<br>R10<br>R11	Refer to prescriber<br>Refer to hospital<br>Refer for medication review<br>Other referral required
Provision of information	R12<br>R13<br>R14<br>R15	Education or counselling session<br>Written summary of medications<br>Commence dose administration aid<br>Other written information
Monitoring	R16<br>R17	Monitoring. Non-laboratory<br>Monitoring. Laboratory test
Other	R18	No recommendation necessary
